# Min-entropy estimation for semiconductor superlattice true random number generators

**DOI:** 10.1038/s41598-022-06815-2

**Published:** 2022-02-22

**Authors:** Jing Liu, Jianguo Xie, Lu Chao, Han Wu, Peng Ding, Xiaoming Chen, Huamin Feng

**Affiliations:** 1 Beijing University of Posts and Telecommunications, Beijing, 100876 China; 2 Beijing Electronic Science and Technology Institute, Beijing, 100070 China; 3 Suzhou Institute of Nano-Tech and Nano-Bionics, Chinese Academy of Sciences (CAS), Suzhou, 215123 China

**Keywords:** Computer science, Statistics, Information theory and computation

## Abstract

Semiconductor superlattice true random number generator (SSL-TRNG) has an outstanding practical property on high-throughput and high-security cryptographic applications. Security in random number generators is closely related to the min-entropy of the raw output because feeding cryptographic applications with insufficient entropy leads to poor security and vulnerability to malicious attacks. However, no research has focused on the min-entropy estimation based on the stochastic model for SSL-TRNG, which is a highly recommended method for evaluating the security of a specific TRNG structure. A min-entropy estimation method is proposed in this paper for the SSL-TRNG by extending the Markov stochastic model derived from the memory effects. By calculating the boundary of the transition matrix, the min-entropy result is the average value of each sample (1 bit) is 0.2487. Moreover, the experimental results show that the estimator is accurate enough to adjust compression rate dynamically in post-processing to reach the required security level, estimating entropy on the fly rather than off-line.

## Introduction

Random number generators play a vital role in the security of communication systems and are basic primitive in cryptographic applications^1,2^. There are two main categories of random number generators according to the random numbers generated whether to rely on deterministic algorithms: pseudo-random number generators (PRNGs) and true random number generators (TRNGs). The random number is used in various cryptographic application scenarios^3^, such as keys, digital signatures, padding values, etc. Moreover, many cryptographic algorithms, protocols, and even hardware attack countermeasures depend on the security of the random number generator in the final analysis.

PRNG generates random numbers based on algorithmic processes and a short true random seed. PRNGs are usually faster than TRNGs and provide highly unbiased random numbers, but the numbers are predictable. If the random number generated by RNGs has lower unpredictable, then the random number can be easily guessed and will cause an attack on the cryptographic system^4–6^. In this way, an unpredictable random sequence is essential to the security of the entire cryptographic application. Therefore, numerous efforts have been devoted to developing TRNGs that generate random numbers via physical noise. TRNGs are attractive alternatives because they provide randomness based on physical phenomena, such as thermal noise, radiation, radio noise, or noise from sensors in mobile devices^7–12^. In addition, TRNGs also provide a solution to the problem of having insufficient entropy^13^. However, the direct output of the available source is biased, so the post-processing part of the source is essential to generate a full entropy sequence^14,15^. Thus, it is imperative to evaluate the entropy of the source.

*Entropy* is a measurable physical property correlated with a state of disorder, randomness, or uncertainty. It reflects the uncertainty by predicting a value prior to observation-the more significant the amount of entropy, the greater the uncertainty in predicting the value of observation^16^. The information entropy contained in the random numbers generated by TRNG is widely used, which can effectively measure the true randomness of TRNG and become an evaluation standard of TRNG security. The post-processing called randomness extraction in TRNG aims to produce shorter and almost uniformly distributed random sequences. The scientific literature provides us the method of how many random bits are extracted from the entropy source, which adjusts the parameter of the extractor^17–19^. Shannon entropy often leads to overestimating total security when applied to a weak source and causes real-world attacks^20,21^. Thus, it fulfills to know the min-entropy to construct a good random number generator, which is a very conservative method and provides the lower bound extracted from the entropy source.

However, estimating entropy is a challenging task since the output distribution of the entropy source is usually unknown, and the common assumptions made on the entropy source may not match the actual situation. At present, theoretical entropy estimation and statistical entropy estimation are the mainstream methods to estimate the entropy. References^22–25^ introduced the theoretical proof of TRNG safety obtained from a reasonable random model. However, making appropriate assumptions is already complicated, not to mention that some TRNG structures do not even have apposite stochastic models^26,27^. Relatively, statistical entropy estimation treats various types of TRNGs as black boxes for statistical testing and still based on the idea of entropy estimation, which can solve some problems that TRNGs cannot quantify by modeling entropy estimation. According to the ISO/IEC 18031^28^ and AIS 31^29^ standards, it is recommended to use theoretical entropy estimation to evaluate the quality of TRNG.

Semiconductor superlattices (SSL) is an all-solid-state electronic device periodically grown by two semiconductor materials with matching lattice^30^. In 1996, Zhang et al.^31^ first observed the chaos current oscillation in a lightly doped and weakly coupled GaAs/AlAs superlattice under a DC bias voltage. However, the chaos oscillation phenomenon only can be observed in a limited temperature range. In 2012, Huang et al.^32^ proposed to use GaAs/$\hbox {Al}_{0.45}$ $\hbox {Ga}_{0.55}$As material instead of GaAs/AlAs to grow semiconductor superlattice and successfully observed chaos oscillation phenomenon at room temperature experimentally. Many scholars have confirmed that the SSL is an ideal entropy source by exploring the structure of the GaAs/$\hbox {Al}_{0.45}$ $\hbox {Ga}_{0.55}$As SSL and the large-amplitude chaos current oscillation generate truly random numbers^33–35^. Moreover, the high-throughput embedded system of semiconductor superlattice true random number generator (SSL-TRNG) was reported recently^36^. SSL-TRNG is very practical, and the random numbers generated can be used as a key in high-end security cryptographic applications to ensure security. However, no research has focused on the security analysis based on the stochastic model for SSL-TRNG.

In this paper, for the first time, we introduce the Markov stochastic model derived from the memory effects of SSL-TRNG and its use for min-entropy estimation in realistic conditions. First, the lower bound of the min-entropy is obtained by computing the boundary of the transition matrix at a high confidence level. Then we design simulations and experiments to verify the theoretical conclusions. By computing bounds on the transition matrix, the min-entropy result is 0.2487 on average per sample (1 bit). Therefore, more SSL-SKD output bits can significantly increase the speed of random number generation and the efficiency of entropy utilization to ensure sufficient entropy through the method proposed in this paper. Moreover, we demonstrate that the estimator is effective enough to support online estimation.

## Results

### Entropy source

The chaotic oscillation phenomenon of SSL can be used to generate random bits at high speed and enough entropy, which has attracted considerable interest recently^33–35^. Under specific offset voltage, the SSL is an ideal non-linear dynamic system with one-dimensional multi-degree-freedom. Its non-linear characteristic comes from the negative differential conductance phenomenon is caused by electrons forming cascade resonance tunneling through quantum wells^31,32^. Since quantum mechanics is extremely sensitive to specific nanostructures in SSL, random fluctuations affect the atomic level during the growth process result in the unique and unpredictable nanostructures of SSL devices. When the static field domain is subject to external interference, the SSL exhibits a transient chaos phenomenon^37^, sensitive to slight differences in input signals. At the same time, it has a memory effect^38^ due to the charge storage of the quantum well. Under continuous input signal excitation, experimental observations show that at the specific moment, the output of a superlattice device is not only related to the current excitation but also related to the dynamic system state caused by the accumulation of historical inputs. Besides, the output bandwidth of the SSL can reach 500 MHz due to the high-frequency chaos oscillation.

As it turned out, the SSL combines with high-throughput and high-security as the entropy source to generate random numbers has the following application advantages: (1) The random number is generated and derived internally by the physical structure and cannot be cloned mathematically and physically. (2) The SSL devices can mass-production parallelly in standard semiconductor manufacturing processes. (3) The SSL can operate above room temperature and resist environmental fluctuations and human interference. (4) The SSL devices are low in cost and simple in application mechanism, which can easily implement electronically.

### SSL-TRNG principle

**Figure 1:**
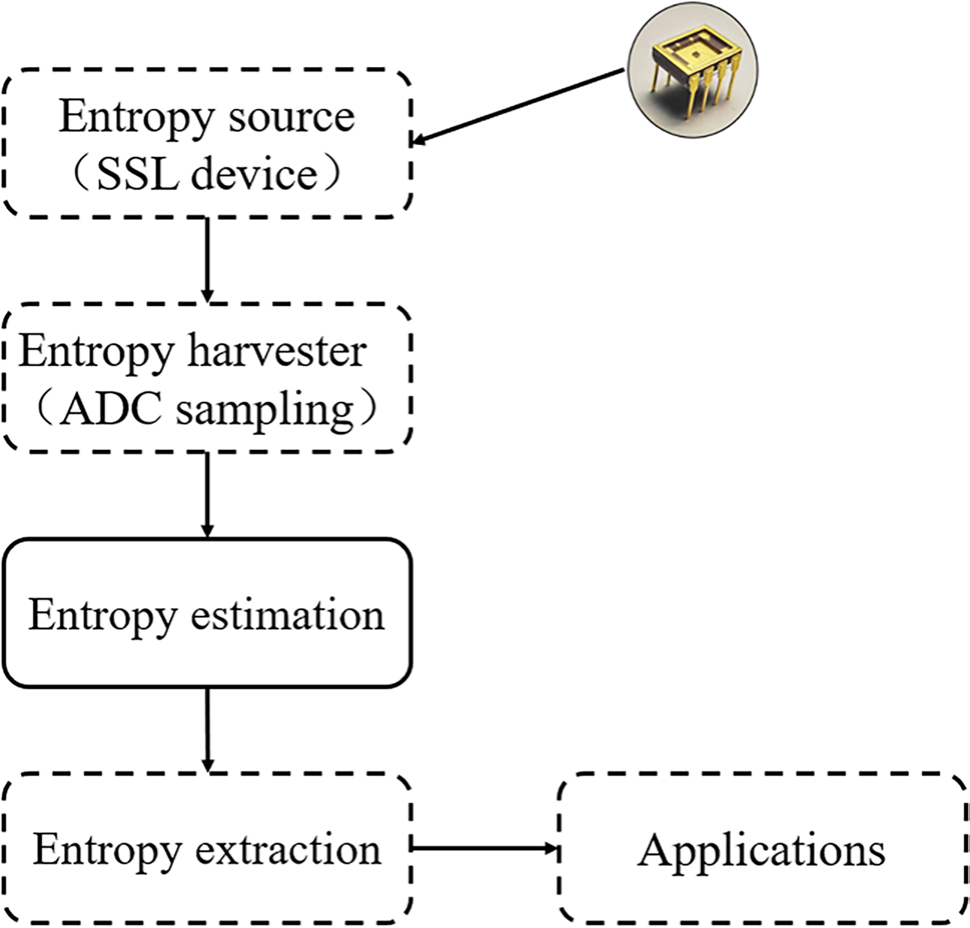
Core architecture of SSL-TRNG.

Figure 1 shows the architecture of the SSL-TRNG. The SSL device exhibits excellent performance as an entropy source to generate a random sequence. The TRNG system generally is composed of the three fundamental components: entropy source, entropy harvester, and entropy extraction^39^. Entropy estimation, adding to the components of SSL-TRNG, and providing security guarantee and anomaly detection to applications.

The entropy harvester is a generalized mechanism that samples the original waveform output from the entropy source and converts it into a binary sequence. Its implementation efficiency depends on the efficiency of the selected entropy source. At first, the output of the SSL device will be digitized since it is an analog waveform. Then, through the analog-to-digital converter (ADC) digitization process, the chaotic current signal can be sampled and quantized into the original random sequence.

The entropy estimation gives how much entropy is contained in the original number sequence and provides parameters for entropy extraction. Moreover, the online entropy estimation mechanism finds out the running defects of the system in time and ensures robustness.

The entropy extraction, also known as randomness extractor, aims to convert the original random sequence from harvester into shorter and almost uniformly distributed random sequences. Numerous extraction methods such as the XOR method, Von Neumann extractor, and least significant bit (LSB) function are used widely^40,41^. Although these schemes are simple to implement, they may fail to correct the deviation and cause high entropy loss^42^. In the following narrative, universal hash functions will be our scheme to provide information-theoretic security^43^. The entropy extraction stage is compressive, and the full entropy random sequence will be obtained through this process.

### Time complexity and space complexity

In the entropy estimation algorithm proposed in this paper, the most time-consuming is to obtain the *P* matrix from the original sequence. The process of calculating the *P* matrix requires a double “*for*” loop, where the length of the original sequence determines the number of the outer “*for*” loops and the inner is the quantized state space size. Obviously, in this experiment, the quantized state space size is 2. Then, get the $T(n)=O(n)$.

In the program operation, the temporarily stored data includes the original data sequence and the transition matrix. The size of the matrix will calculate by bit quantization of the data. In this experiment, 1-bit quantization is 4, and then get the $S(n)=O(n)$.

From the above discussion, the algorithm has linear time and space complexity. It has obvious advantages for realizing online entropy estimation.

### Estimation results

Using the method of estimating the min-entropy proposed in this paper, we conducted multiple sampling tests on the original data sequence generated by SSL-TRNG, and each sample contained 1,000,000 samples. The obtained min-entropy results are shown in Table 1. Table 1 also lists the calculation results of the transition matrix in estimating the min-entropy and the matrix boundary when the confidence level is 95%. Similarly, using the Markov Model of NIST to estimate the min-entropy of original output sequence, the results are listed in the right column of this work.

**Table 1:** The results in this paper and compare with the Markov model in NIST SP 800-90B.

Data	P-matrix	M-matrix	Entropy (per 1 bit)	
			This work	Markov
1	$$\begin{bmatrix} 0.90899 &{} 0.09101 \\ 0.14542 &{} 0.85458 \end{bmatrix}$$	$$\begin{bmatrix} 0.91009 &{} 0.09210 \\ 0.14678 &{} 0.85583 \end{bmatrix}$$	0.24871468	0.39214177
2	$$\begin{bmatrix} 0.91396 &{} 0.08604 \\ 0.15002 &{} 0.84998 \end{bmatrix}$$	$$\begin{bmatrix} 0.91503 &{} 0.08712 \\ 0.15141 &{} 0.85127 \end{bmatrix}$$	0.24388940	0.36306248
3	$$\begin{bmatrix} 0.92223 &{} 0.07777 \\ 0.16478 &{} 0.83522 \end{bmatrix}$$	$$\begin{bmatrix} 0.92323 &{} 0.07880 \\ 0.16628 &{} 0.83666 \end{bmatrix}$$	0.24874552	0.30524206
4	$$\begin{bmatrix} 0.90053 &{} 0.09947 \\ 0.13527 &{} 0.86473 \end{bmatrix}$$	$$\begin{bmatrix} 0.90161 &{} 0.10059 \\ 0.13657 &{} 0.86599 \end{bmatrix}$$	0.25406888	0.45089208
5	$$\begin{bmatrix} 0.90071 &{} 0.09929 \\ 0.15131 &{} 0.84869 \end{bmatrix}$$	$$\begin{bmatrix} 0.90177 &{} 0.10038 \\ 0.15265 &{} 0.84999 \end{bmatrix}$$	0.26065681	0.40865878
6	$$\begin{bmatrix} 0.91822 &{} 0.08178 \\ 0.17373 &{} 0.82627 \end{bmatrix}$$	$$\begin{bmatrix} 0.91922 &{} 0.08281 \\ 0.17523 &{} 0.82770 \end{bmatrix}$$	0.24377304	0.30452169
7	$$\begin{bmatrix} 0.91792 &{} 0.08208 \\ 0.17631 &{} 0.82369 \end{bmatrix}$$	$$\begin{bmatrix} 0.91892 &{} 0.08311 \\ 0.17781 &{} 0.82514 \end{bmatrix}$$	0.24473328	0.30150534
8	$$\begin{bmatrix} 0.90824 &{} 0.09176 \\ 0.15874 &{} 0.84126 \end{bmatrix}$$	$$\begin{bmatrix} 0.90927 &{} 0.09283 \\ 0.16014 &{} 0.84261 \end{bmatrix}$$	0.25407353	0.36572432
9	$$\begin{bmatrix} 0.91396 &{} 0.08604 \\ 0.17018 &{} 0.82982 \end{bmatrix}$$	$$\begin{bmatrix} 0.91497 &{} 0.08709 \\ 0.17165 &{} 0.83122 \end{bmatrix}$$	0.24932592	0.32468331
10	$$\begin{bmatrix} 0.90376 &{} 0.09624 \\ 0.14035 &{} 0.85965 \end{bmatrix}$$	$$\begin{bmatrix} 0.90483 &{} 0.09734 \\ 0.14168 &{} 0.86093 \end{bmatrix}$$	0.25284791	0.42455133

According to the results of entropy estimation in Table 1, the min-entropy (per 1 bit) of the original output sequence of the superlattice device is 0.2487 bit (this work) and 0.3641 bit (Markov model). This work finds the lower bound of the min-entropy based on the Markov method, so the entropy estimation results are more accurate, which can also reflect in the Table 1. The results also indicate the upper limit of the compression rate of the entropy extractor. In addition, it can find that the result of min-entropy has less fluctuation, which can reflect the stability of the random number system of the superlattice device to a certain extent.

### Statistical test

There is a recognized and accepted standard for statistical testing randomness, which is the statistical test suite 800-22 from the National Institute of Standards and Technology (NIST)^44^ contains 15 sub-test items.The NIST standard requires that the length of the sequence to be tested should be at least 1Mbit, and their uniformity judge by checking the distribution of the P-values. The judgment result gives by the P-value $P_T$ and the proportion $\sigma$. In this experiment, 1000 bitstreams with a length of 1Mbit used for the NIST statistical test under the significance level 0.01. Then, the P-value $P_T$ should be greater than 0.0001, and the proportion $\sigma$ should be greater than 0.98. Table 2 shows the NIST SP 800-22 statistical test results for SSL-TRNG. We conclude that the random numbers generated by SSL-TRNG can pass the evaluation of NIST 800-22, where the parameters of the extractor are determined by the entropy estimation results in this paper.

**Table 2:** The results of NIST statistical test when the significance level is 0.01.

Statistical test	P-value	Proportion	Results
	$P_T>10^{-4}$	$\sigma >0.980$	
Frequency	0.7301	0.983	Success
Block frequency	0.6329	0.995	Success
Cumulative sums	0.8898	0.986	Success
Runs	0.5781	0.994	Success
Longest run	0.2564	0.988	Success
Rank	0.4239	0.993	Success
FFT	0.9061	0.987	Success
Non-overlapping template	0.7575	0.992	Success
Overlapping template	0.9726	0.998	Success
Universal	0.3990	0.991	Success
Approximate entropy	0.4735	0.987	Success
Random excursions	0.1748	0.990	Success
Random excursions Variant	0.5718	0.981	Success
Serial	0.3798	0.997	Success
Linear complexity	0.2659	0.984	Success

## Discussion and conclusions

Entropy is an important metric in secure systems. There are many methods of entropy estimation. In addition to min-entropy, there is Shannon entropy, Rényi entropy, collision entropy, etc. In this paper, the conservative method is used to estimate the min-entropy of sequences generated by semiconductor superlattice to ensure that the SSL-TRNG outputs full entropy random numbers. According to the entropy estimation results and the property of the SSL entropy source, SSL-TRNG can generate full entropy sequences at high speed, which can satisfy the application of one-time pad cipher. At the same time, it can provide random bits for the cryptographic primitive such as symmetric ciphers, public-key cryptography, certificates, signatures, which play a significant role in the blockchain and the Internet of Things to protect core applications and defend against invasion^45–47^.

We collect TRNGs with ADC sampling Oscillate, Optical vacuum fluctuation, Stokes field phase fluctuations, and quantum as entropy sources and show the min-entropy (per sample) and full-entropy throughput (Mb/s) of SSL-TRNG in comparison with that of other TRNGs in Table 3. In terms of security and performance, our work achieves significantly higher entropy bit rates for a given confidence level than the TRNG of ADC sampling Oscillate (33 Mb/s in Ref.^13^), the TRNG of Stokes field phase fluctuations (145 Mb/s in Ref.^48^). The only directly comparable work which offers a min-entropy (Per 1 bit) is Ref.^13^, whose full-entropy throughput is less 46 times than ours. Our total entropy throughput rate is slightly lower than that of quantum TRNG (1770 Mb/s in Ref.^49^), and TRNG, whose entropy source is optical vacuum fluctuation, is (6000 Mb/s in Ref.^50^) four times that of ours.

SSL-TRNG performs well in cryptographic applications with high-security and high speed requirements. Compared with true random number generators, which use other physical entropy sources, SSL-TRNG fully adapts in terms of throughput, frequency, area, etc. Though, SSL-TRNG is easy to implement lightweight and miniaturized hardware. In addition, SSL devices can be mass-produced and are resistant to environmental fluctuations and human interference. They are implemented electronically without the high cost and complex application mechanisms. It achieves the best balance between speed and ease of use.

**Table 3:** Comparison results with other TRNGs.

Work	Entropy source	Min-entropy (per sample)	Full entropy throughput (Mb/s)
Reference^13^	ADC sampling oscillate	0.17 bit / 1 bit	33
Reference^51^	Optical vacuum fluctuation	6.53 bit / 12 bit	6000
Reference^52^	Stokes field phase fluctuations	4 bit / 16 bit	145
Reference^53^	quantum	1.5 bit / 8 bit	1770
This work	SSL	0.2487 bit / 1 bit	1554

SSL-TRNG uses semiconductor superlattices as physical entropy source to generate truly random numbers. And entropy estimation provides a crucial evaluation for the security of SSL-TRNG. In this work, we propose a min-entropy estimation method for the SSL-TRNG and verify its feasibility for the first time. In particular, the stochastic model established using the Markov model as a template heuristically. By looking for the boundary of the Markov transition matrix, get the lower bound of min-entropy under a high confidence level. Through experiments, the average result of min-entropy is 0.2487 per sample (1 bit). In addition, the results also prove that the estimator is accurate enough to dynamically adjust the compression ratio in post-processing to achieve the required security level, estimating entropy instantly instead of offline.

The work of this paper not only provides a security guarantee for SSL-TRNG, but also a new clew for the research of quantifying the SSL physical entropy source. Our future work will be extended by adding experimental samples, expanding the entropy estimation model and in-depth analysis entropy source to this research, further enhancing model selection and parameter optimization for similar entropy estimation problems.

## Methods

### Preliminaries

*Min-entropy* is the most conservative method to measure the unpredictability of a set of sequences.

#### Definition 1

Suppose that the independent discrete random variable *X* takes a value from the finite set $A={x_1,x_2,\ldots ,x_n}$ when $i=1,\ldots ,n$, the min-entropy with probability $P_r (X=x_i )=p_i$ is

1 $$\begin{aligned} H=\min _{ 1 \le i \le k}(-log_2 p_i) =-log_2 \max _{ 1 \le i \le k}p_i . \end{aligned}$$

From the previous discussion, the output sequence from the SSL-TRNG entropy source has memory effects. The current output is not only related to the current excitation but also the historical input. The dependency between the output sequence is the most complex complication to address^54^. It should think whether it is feasible to solve this difficulty by accepting a simple output-dependent model and analyzing the model, but in fact, it is impractical or impossible to obtain an accurate stochastic model of the output sequences.

The Markov model^55^ is a typical example of data dependence: the next output state of the *N*-order Markov process depends on the previous *N* output states. Heuristically, we use the Markov model as a template and establish a stochastic model for the output sequence of the SSL-TRNG entropy source. Therefore, the dependence of the output sequence is limited to the Markov process.

#### Definition 2

The Markov process defines by three elements:

1. State space *X*. *X* is a set containing all states.
2. Transition matrix *P*. The elements in *P* are defined as
  2 $$\begin{aligned} P_{ij}=p\bigg (x^{(t+1)}=j|x^{(t)}=i\bigg ), \end{aligned}$$
  which means the transition probability from the current state *i* to the next state *j* is $P_{ij}$.
3. Initial state distribution $p(x^{(0)})$. The meaning is that when $t=0$, *x* takes the corresponding probability of any possible state in the state space.

A stochastic process $\{X_n \}_{n \in {\mathbb {N}}}$that takes values from a finite set *A* is called a *first-order Markov chain*^56^, if

3 $$\begin{aligned} P_r&\bigg (X_{n+1}=x_{n+1}|X_n=x_n,X_{n-1}=x_{n-1},\ldots ,X_0=x_0\bigg ) = P_r\bigg (X_{n+1}=x_{n+1}|X_n=x_n\bigg ), \end{aligned}$$

for all $n \in {\mathbb {N}}$ and all $x_0,x_1,\cdots ,x_k \in A$ . The initial probability $p(x^{(0)})$ of the chain are $p_i=P_r (X_0=i)$, whereas the transition probabilities $P_{ij}$ are $P_r (X_{n+1}= j \vert X_n=i)$.

#### Definition 3

In a *d*-th order Markov chain, the transition probabilities satisfy

4 $$\begin{aligned} P_r&\bigg (X_{n+1}=x_{n+1}|X_n=x_n,X_{n-1}=x_{n-1},\ldots ,X_0=x_0\bigg ) = P_r\bigg (X_{n+1}=x_{n+1}|X_n=x_n,\ldots ,X_{n-d}=x_{n-d}\bigg ). \end{aligned}$$

#### Definition 4

The min-entropy of a Markov chain with length L is defined as

5 $$\begin{aligned} H=-log_2\bigg (\max _{i_1,\ldots ,i_L } p_{i_1}\prod _{j=1}^L p_{i_ji_{j+1}}\bigg ). \end{aligned}$$

### Min-entropy estimation of TRNG

The entropy estimation method of TRNG includes two processes: establishing a stochastic model and estimating entropy^51^. First, assumptions are made about the entropy source of the TRNG based on the noise model, such as the noise source obeys independent normal distribution. Then, the process of converting noise sources into random bits describes in mathematical language according to the proposed hypothesis and the working principle of TRNG, which is to establish a stochastic model. Finally, the probability distribution of the output can be calculated and estimated the entropy of the TRNG according to the established random model.

As far as we know, lots of work has done to establish stochastic models and estimate entropy for various TRNGs. Generally, TRNGs have their corresponding stochastic models, though some stochastic models are generic and adapt to several generators^25^. Specifically, Refs.^23,24^ investigate models through the evolution of phase, and Refs.^22,52,53,57^ through the time for elementary oscillator-based TRNG (EO-TRNG). The chaos-based TRNGs use ADC to build chaotic circuits^58^ or sample chaotic signals to generate random sequences^26,27^. Under the absence of corresponding stochastic models, the theoretical entropy sufficiency cannot guarantee. The NIST Special Publication 800-90B^55^, whose latest version was published in January 2018, is a typical representative of entropy estimation. Its specific content includes estimating the entropy source’s min-entropy and providing a standard for designing and testing the entropy source. Reference^59^ proposes using neural network technology to solve the min-entropy estimation problem, which provides a new idea for entropy estimation. By extending an existing model and the multi-bit ADC output, Ref.^13^ obtain the lower bound of the entropy for the ADC sampling-based TRNG. Ref.^42^ presents a method for maximizing the conditional min-entropy of the random sequence generated by quantum-to-classical-noise ratio. To address the limitations about the entropy source outputs may be dependent and the distribution of random variables may change over time, Ref.^56^ proposes alternative methods for estimating the entropy in each output from an entropy source based on concepts from machine learning.

### Stochastic model of SSL-TRNG

Suppose that $X(t)=\{x_1,x_2,\ldots ,x_L\}$ are the sampling output sequence of the SSL-TRNG entropy source and the length is *L*. Further, suppose that *X*(*t*) is a Markov process with the initial state distribution $p(x^{(0)})$ and the transition matrix is $P \in [0,1]^{n \times n}$,

6 $$\begin{aligned} P=\begin{bmatrix} P_{11} &{} \cdots &{}P_{1n} \\ &{} \vdots &{} \\ P_{n1} &{} \ldots &{} P_{nn} \end{bmatrix}, \end{aligned}$$

where the *X*(*t*) determines the $p(x^{(0)})$ and the matrix *P*. In $X(t)=\{ x_1,x_2,\ldots ,x_L \}$, we count the frequency of $x^{(0)}$ to estimate $p(x^{(0)})$ and each state transitioning to other states to estimate $P_{ij}$. Obviously, the size of the *X*(*t*) affects their accuracy directly because some infrequent transitions may not appear in the *X*(*t*) data set.

Therefore, the min-entropy of *X*(*t*) can be defined as

7 $$\begin{aligned} H_\infty \bigg (P,p(x^{(0)}),n\bigg )&=\min _{i_1,\ldots ,i_L } -log_2 {\mathbb {P}} \bigg [X_1=x_1 \bigcap \ldots \bigcap X_L=x_L\bigg ] \nonumber \\&= \min _{i_1,\ldots ,i_L } -log_2\bigg (p_{i_1}\prod _{j=1}^L P_{i_ji_{j+1}}\bigg ). \end{aligned}$$

In the Markov process, accurately estimating the transition probability matrix is vital for estimating the entropy. In this case, if we overestimate the transition probability, the min-entropy will be underestimated. However, lots of tests will minimize the possibility.

According to the Eq. (7), $P_{ij}$ is the only variable. Then the minimum bound of the min-entropy $H_\infty$ by finding the maximum bound of the transition matrix *P*. Suppose there is a matrix *M* such that $M_{ij} \ge P_{ij}(i,j=1,\ldots ,n)$, then $H_\infty (M,p(x^{(0)}),n)\le H_\infty (P,p(x^{(0)}),n)$ by the monotonic decline of the $-log_2$ function^54^.

So how to get such a matrix *M*? Suppose the state *i* from *X*(*t*), and the transition probability $P_{ij}$ from state *i* to state *j*, where $i,j = 1,\ldots ,n$. We choose a value $m_{ij}$ and define the confidence interval $[0,m_{ij}]$, so that our choice satisfies the confidence level $\alpha :{\mathbb {P}}[M_{ij}\le m_{ij}\vert p_i,p_{ij}]\ge \alpha$.

The interval with the confidence level $\alpha$ is obtained by calculating the probability that more transitions are expected to be observed than actual. Similarly, we can define $m_{ij}$ in terms of the observed proportion:

8 $$\begin{aligned} m_{ij}=\min \bigg \{1,\frac{p_{ij}}{p_i} +\epsilon \bigg \}, \end{aligned}$$

where

9 $$\begin{aligned} \epsilon =\sqrt{\frac{1}{2p_i}log\bigg (\frac{1}{1-\alpha }\bigg )}. \end{aligned}$$

Then, Hoeffding’s inequality limits the error of matrix M within the prescribed confidence.

In this way, the bound of the transition matrix $M\in [0,1]^{n \times n}$ the value of $m_{ij}$ is calculated by Eq. (8):

10 $$\begin{aligned} M=\begin{bmatrix} m_{11} &{} \cdots &{}m_{1n} \\ &{} \vdots &{} \\ m_{n1} &{} \cdots &{} m_{nn} \end{bmatrix}. \end{aligned}$$

With the probability $\alpha ^{\min \{n^2,N\}}$, the calculation of the min-entropy for the matrix *M* is the lower bound for the min-entropy of the *N* outputs of the superlattice physical entropy source: $H_\infty (M,p(x^{(0)}),n)\le H_\infty \bigg (P,p(x^{(0)}),n\bigg ).$

**Example**: Let *X* be (1, 0, 0, 0, 1, 0, 1, 1, 1, 1, 0, 0, 1, 0, 1, 0, 0, 1, 0, 0, 1, 0, 1, 0, 1, 0, 0, 0). So that the initial state distribution $p_0=4/7$, $p_1=3/7$ and the transition matrix $$P=\begin{bmatrix} 7/27 &{} 8/27 \\ 9/27 &{} 3/27 \end{bmatrix}$$. Calculate the min-entropy of *X* by Eq. (7), the value of $H_\infty (P,p(x^{(0)}),n)$ equal to 0.08 bit / 1 bit approximately. For the purpose of this example, suppose that $\alpha =0.05$, then $\epsilon _0=0.14$, $\epsilon _1=0.16$. Calculate $m_{ij}$ by Eq. (8), the bound of the transition matrix $$M=\begin{bmatrix} 0.5937 &{} 0.6585 \\ 0.9378 &{} 0.4192 \end{bmatrix}$$. Then the lower bound for the min-entropy $H_\infty (M,p(x^{(0)}),n)\approx$ 0.03857 bit / 1 bit.
